# Low-call-rate SNPs and presence–absence variation identified in the rice pan-genome can improve genomic prediction of rice gene bank accessions

**DOI:** 10.1007/s00122-025-05080-x

**Published:** 2025-11-07

**Authors:** Lukas Krusenbaum, Matthias Wissuwa

**Affiliations:** Institute of Crop Science and Resource Conservation (INRES), University of Bonn, Bonn, Germany

## Abstract

**Key message:**

Substantial improvements in genomic prediction accuracy for rice gene bank accessions were achieved by incorporating SNPs of low call rate identified in a recently published rice pan-genome.

**Abstract:**

Introduction of useful genetic variation to breeding populations is a key factor in achieving genetic gain in crop breeding. However, identifying donors from genetic diversity stored in gene banks requires extensive phenotyping, which is not feasible for many traits of interest. Genomic prediction (GP) of phenotypic values has been proposed to overcome this phenotyping bottleneck. A key challenge for GP is the identification of appropriate markers representative of genetic variation causal for phenotypes. Here we report on utilizing single nucleotide polymorphisms (SNPs) from the core and dispensable genomes of a rice pan-genome resource comprising 16 reference sequences. Using a published pan-genome graph, we identified SNPs within structural variations of the dispensable genome. In this SNP set, SNPs of low call rate (CR) were common. Presence–absence variation (PAV) of these SNPs was associated with subpopulation structure, indicating that SNP absence reflects on underlying sequence PAV rather than being solely due to technical errors in SNP detection. To incorporate these SNPs in GP models, we employed modified encoding, retaining information of PAV and nucleotide variation by one-hot encoding (OHE). Adding these to SNP matrices increased prediction accuracies of GP for some traits and subpopulations. Improvements could largely be attributed to the inclusion of PAV. Our results show that the traditional approach of applying strict CR filters to SNPs located in the dispensable genome disregards potentially valuable genetic information not in linkage with SNPs of high CR. The proposed strategy provides a straightforward way to enhance GP performance in rice gene bank accessions.

**Supplementary Information:**

The online version contains supplementary material available at 10.1007/s00122-025-05080-x.

## Introduction

Breeding relies on a constant stream of new and beneficial genetic material for a variety of traits, from agronomic and stress tolerance to quality traits. While ample genetic diversity is stored in gene banks, utilization of this material requires extensive phenotyping efforts that are not feasible for many traits. Overcoming the phenotyping bottleneck is crucial for the full utilization of gene bank potential (Furbank and Tester 2011; McCouch et al. 2013).

Genomic prediction (GP) was first described in 2001 to estimate genomic estimated breeding values (GEBVs) (Meuwissen et al. 2001) and has been employed successfully as a breeding tool for a large number of crops. In the context of gene bank phenomics, GP has been discussed as a tool to leverage phenotypic data in large gene bank collections, especially for traits that are cost-intensive to phenotype. This development is further fueled by decreasing costs of genetic markers overcompensating for reductions in the accuracy of GEBVs compared to classical breeding values (Yu et al. 2016; El Hanafi et al. 2023). For example, GP was used to identify new donors from rice gene bank accessions for improving adaptation to low soil fertility (Tanaka et al. 2021), for elevated grain zinc (Rakotondramanana et al. 2022), or for prediction of agronomic performance under drought for wheat gene bank germplasm (Crossa et al. 2016). However, inferences from genotype to phenotype remain challenging due to the complex nature of genotypic variation in plants.

Within the 3 k rice genomes project, short-read sequencing data (14 × average sequencing depth) of 3024 diverse rice gene bank accessions representing all major subpopulations of *Oryza sativa* are publicly available (Li et al. 2014). SNP data for this have previously been generated by mapping these short reads to a single reference sequence (Nipponbare). Using these SNP data, four main subpopulations have been established for *O. sativa* (*aromatics*, *aus*, *indica*, and *japonica*), with *indica* and *japonica* further clustering into *indica1A*, *indica1B*, *ind2*, *ind3,* and *tropical*, *subtropical*, and *temperate japonicas,* respectively. Within the 3 k rice genomes project, admixed accessions as well as admixed *indicas* and admixed *japonicas* can be identified (Wang et al. 2018).

It has become widely acknowledged that a single reference sequence may not be adequate to represent sequence diversity in a species (Bayer et al. 2020; Shi et al. 2023), and plant pan-genomes have been published for several crop species, including rice (Zhou et al. 2020; Qin et al. 2021; Sun et al. 2017), barley (Jayakodi et al. 2024), and tomato (Zhou et al. 2022; Li et al. 2023). Within these pan-genomes, large sequence variations can be detected. These include inversions, translocations, copy number variations (CNV), and presence–absence variations (PAV). The latter is an extreme form where large stretches of sequence may be completely absent in an individual. (Saxena et al. 2014) Using the concept of the pan-genome, genomic regions of a species can be separated into a core (common to all individuals) and a dispensable fraction (absent in some individuals). Having multiple reference sequences available per species has been discussed as a valuable tool to identify novel single nucleotide polymorphisms (SNPs) within this dispensable genome (Hurgobin and Edwards 2017).

Recently an additional 15 near gap-free reference sequences have been made publicly available, representing the wider gene pool of cultivated rice. In addition to the original Nipponbare (NB) reference sequence representing the *temperate japonica* subspecies (International Rice Genome Sequencing Project 2005; Hu et al. 2018; Kawahara et al. 2013), the 15 new reference sequences include Minghui63 (MH63, *admixed indica*), ZhenShen97 (ZS97, *indica1A*) (Zhang et al. 2016), N22 (*aus*) (Stein et al. 2018), and an additional 12 reference sequences (Zhou et al. 2020) representing the subpopulation structure of cultivated rice. Short reads from the 3 k rice genomes project were then mapped to these additional 15 reference sequences, resulting in 16 SNP sets that are publicly available (Zhou et al. 2024). Within this newly published pan-genomic resource, a total of 36.6 Mb of genomic sequence absent from the NB reference was identified, including ~ 2.3 million novel SNPs (Zhou et al. 2024). Additionally, these 16 sequences were used to build a pan-genome graph (Zhou et al. 2023).

Pan-genome graphs are employed to represent the sequence diversity in a species. They are generated using a reference sequence as a backbone to which new sequences are consecutively aligned, storing large variations to the previously investigated sequences. This method provides a graph structure that captures larger sequence variations relative to the backbone sequence. Vertices of this graph represent stretches of sequence, and an individual sequence used to build the graph can now be described as a pass through the graph. (Wang et al. 2023) By looking at sequence variation represented in the pan-genome graph, one can identify structural variations belonging to the dispensable genome that are not present in the backbone sequence. Previous work in rice identified a large dispensable genome for which variation is clustering with population structure (Sun et al. 2017; Wang et al. 2018). When looking at SNP variation identified by mapping short reads to a reference sequence, the choice of the reference sequence should not matter for all SNPs located in the core genome, as these sequences are present in all accessions. Novel SNPs that have not been identified by mapping to a single reference sequence are to be found in the dispensable fractions of the genome (Hurgobin and Edwards 2017).

Efforts to utilize genetic variation in the dispensable genome have shown that information on the presence and absence of structural variants can improve genome-wide association studies (GWAS) or predictions of phenotypes in several crop species (Zhou et al. 2022; He et al. 2023; Liu et al. 2024; Yan et al. 2023; Yan et al. 2024; Qin et al. 2021). Even prior to the availability of pan-genomes, it was demonstrated that the dispensable genome contained important stress tolerance genes, and the *Pup1* and *Sub1* loci conferring tolerance to phosphorus deficiency and submergence, respectively, are good examples from rice (Heuer et al. 2009; Xu et al. 2006). So far, novel SNPs identified by the newly released 3 k pan-genomic resource have not been used for GP, and methods on how to select and incorporate SNPs from the dispensable sequences for GP need to be optimized.

Typically, biallelic markers can be encoded as continuous variables by alternative allele counts. When these are used as predictors in multiple regression models, genetic effect parameter estimates are based on regressions of the alternative allele counts to the phenotype, capturing the additive effect of the alternative allele. Calling SNP markers from short-read mapping or SNP chips can cause missing SNP calls (e.g., through insufficient coverage or errors in the hybridization assay). To ensure compatibility with models, a typical strategy to handle such missing values is to filter SNPs by their call rate (CR), defined as the frequency of non-missing values. The remaining missing values are then imputed, under the assumption that they result from technical errors in the genotyping pipeline (Rakotondramanana et al. 2022; Yuan et al. 2020; Zhang et al. 2019; Sang He et al. 2022; Tanaka et al. 2021). However, for SNPs residing within PAV, true absence has to be expected by definition for at least some portion of the accessions studied, and this absence (and presence) may represent valuable information. Imputing such missing SNPs would potentially mask such information (Della Coletta et al. 2021), while applying strict CR filters would eliminate a large portion of the dispensable genome and the information therein. Previous studies have used presence–absence variation (PAV) of SNPs of low CR from SNP chip data in GWAS (Gabur et al. 2018, 2020; Vollrath et al. 2021b, 2021a) and GP (Weber et al. 2023). While prediction of phenotypes using presence–absence variation of SNPs was possible, no improvement of prediction accuracies compared to solely employing SNPs of high CR was observed in the above studies for maize and canola.

Encoding SNPs of low CR solely by PAV omits information on nucleotide variation at the SNP. Retaining information on the nucleotide would require accepting three possible alleles for a SNP. Variation of such a SNP would therefore become categorical, and straightforward encoding as a continuous variable by alternative allele counts is no longer possible. A typical approach of encoding categorical variables for machine learning models is one-hot encoding (OHE). Instead of encoding SNP variation by alternative allele counts, additional columns are added to the marker matrix indicating the genotypic state of a diploid individual at a specific locus. For example, genotypic states (NA, A_1_A_1_, A_1_A_2_, A_2_A_2_) would be encoded as (1 0 0 0), (0 1 0 0), (0 0 1 0), and (0 0 0 1), respectively.

In the present study, we are investigating the potential to improve GP of rice gene bank accessions by utilizing SNPs identified in pan-genomes. A particular question addressed is whether the absence of SNPs due to the potential absence of the underlying sequence can be employed as a reasonable predictor and whether distinguishing the allelic state of partially absent SNPs through OHE can further improve prediction accuracies. To that effect, we have used the 3 k pan-genome data set of rice to: 1) investigate SNP variation found in the dispensable fraction of the rice genome by selecting SNPs using a pan-genome graph. We hypothesize that SNPs within the dispensable genome would show a substantial amount of presence–absence variation, requiring alternative strategies of handling such SNPs; 2) to modify SNP encoding in order to capture patterns of missingness while retaining information on the nucleotide state through OHE; and 3) to compare the power of different SNP sets derived from standard SNP filtering or encoding as PAV and OHE in GP of rice gene bank accessions. We hypothesize that SNPs in the dispensable genome of particularly low CR will be especially useful for GP.

## Methods

All analyses are based on the 16 reference sequences publicly available from the 3 k rice genomes project (International Rice Genome Sequencing Project 2005; Hu et al. 2018; Kawahara et al. 2013; Zhang et al. 2016; Stein et al. 2018; Zhou et al. 2020). These sequences comprise of: Nipponbare (NB; *temperate japonica*), Minghui63 (MH63; *admixed indica*), ZhenShan97 (ZS97; *indica1A*), N22 (*aus*), Azucena (AZ; *tropical japonica*), IR64 (*indica1B*), ARC 10497 (ARC; *aromatics*), Larha Mugad (LM; *indica2*), Liu Xu (LX; *admixed indica*), Khao Yai Guang (KYG; *indica3*), Lima (*indica3*), Natel Boro (NABO; *aus*), PR106 (*indica1B*), Ketan Nangka (KN; *tropical japonica*), Chao Meo (CM; *subtropical japonica*), and Gobol Sail (Balam) (GS; *indica2*). All 16 SNP matrices, generated by mapping short reads available in the 3 k genomes projects to the 16 reference sequences, were taken from the 3 k rice genomes project (https://snp-seek.irri.org/). Every SNP matrix consists of ~ 27 million SNPs, of which a total of ~ 2.3 million SNPs have been reported to be previously unidentified by mapping to the Nipponbare (NB) reference sequence (Zhou et al. 2024).

### Baseline SNP matrix

From that resource, a baseline SNP set was generated by filtering the SNPs called against the NB reference by call rate (CR) > 0.8, minor allele frequency (MAF) > 0.05, and heterozygosity < 0.05. To reduce the size of the SNP matrix, SNPs in high linkage disequilibrium (LD) were removed by LD pruning using a sliding window of 50 SNPs and an *R*^2^ threshold of 0.8. All operations were performed in plink2 (Chang et al. 2015). In total, this SNP set included 374,735 SNPs. SNPs were encoded as (0 = homozygous reference, 1 = heterozygous, 2 = homozygous alternative). Missing values were imputed by a mean value. This SNP set was used for reference, to compare to a “standard” approach of selecting SNPs for GP. Namely, calling SNPs from mapping short reads to a single reference sequence, applying strict CR filters, and imputing remaining missing values. We will refer to this SNP set as BASELINE for all further reference. To investigate the effects of the number of SNPs in the BASELINE set on prediction accuracies, variations of BASELINE were generated. For these, the *R*^2^ threshold during LD pruning was increased to 0.9 and 0.95, respectively. An overview of the number of SNPs remaining in the resulting SNP matrices is given in Supplementary Table 2.

### Selection of pan-genomic SNPs

For each of the other (non-NB) 15 reference sequences, all remaining (non-NB) SNP sets were filtered by MAF > 0.05, CR > 0.05, and heterozygosity < 0.05 in all 3 k accessions. This step was performed separately for each set. The low-CR filter was applied to ensure that SNPs within particularly rare dispensable sequences would not be dropped from later analysis. Only biallelic SNPs were retained. This reduced the number of SNPs to ~ 4 million per set. Filtering steps were performed in plink2 (Chang et al. 2015). These sets were only intermediate sets, not used for GP. SNPs within large non-NB vertices of the pan-genome graph were later drawn from these sets.

For SNP selection, the pan-genome graph generated by Zhou et al. (2023) was employed. Vertices from the graph represent stretches of sequences in the pan-genome graph. From information on the source reference sequence and its position within that sequence, SNPs identified within a vertex were selected. By selecting only vertices from reference sequences other than the backbone (NB) sequence, it was ensured that selected vertices represent SVs not present in the NB sequence. SNPs along vertices could be selected from intermediate SNP sets described above. As the number of SNPs identified in all vertices was too large to reasonably employ all such SNPs in GP, the analysis was restricted to large vertices (> 5,000 bp), assuming that SNPs in smaller vertices would more likely be in linkage with neighboring SNPs. In a first step, to investigate patterns of missingness in SNPs from graph vertices, all SNPs identified were combined into a single matrix. By this, a total of 1,363,607 SNPs were identified. Call rates for all SNPs over the whole 3 k set as well as subpopulations (*aus*, *japonica*, and *indica*) were calculated. Additionally, the number of SNPs from non-NB vertices that were nevertheless present within NB as well as their CR was recorded.

### Encoding of SNPs with low CR

SNPs of CR < 0.8 were encoded in two ways, either as presence–absence variation (PAV) as described by Gabur et al. (2018) and Weber et al. (2023), or through a process similar to one-hot encoding (OHE). PAV encoding assigns a “0” to a present SNP, while a missing SNP is encoded as “2”. In contrast, OHE also retains information on the nucleotide state of SNPs by adding additional columns to the SNP matrix. A SNP matrix with 3 columns and encoded allele counts of the three possible alleles (absence, A_1_, A_2_) was used. The first column encodes presence–absence as in the PAV matrix (0 or 2), whereas columns 2 and 3 inform on the nucleotide. An individual homozygous for the reference nucleotide allele would code (0 2 0), homozygosity of the alternative nucleotide allele would code (0 0 2), and absence would be coded (2 0 0). An individual heterozygous for the nucleotide alleles would code (0 1 1) (see Fig. 2**)**. This deviates slightly from classical OHE, where heterozygosity would be encoded in column three of a four-column matrix as (0 0 1 0). SNP absence would be (1 0 0 0), homozygosity of the alternative allele would be (0 0 0 1), and homozygosity of the reference allele would be (0 1 0 0). As rice is a highly self-fertile crop, heterozygosity is low, and this classical approach may give spurious associations for the third column due to the low frequency of the heterozygous state.

### Generation of pan-genomic SNP matrices

Three pan-genomic SNP matrices were constructed in addition to the BASELINE matrix. Using the intermediate SNP sets, a pan-genome core matrix (PanG-CORE) was generated by selecting all SNPs along non-NB pan-genome graph vertices with CR > 0.8, imputing missing values by mean values, and adding the resulting SNPs to the BASELINE matrix. A PanG-PAV matrix was created by encoding all SNPs with CR < 0.8 along vertices by PAV and adding the resulting SNPs to the BASELINE matrix. Third, a PanG-OHE matrix was created by employing OHE instead of PAV to SNPs with CR < 0.8.

To reduce redundancies in the respective CORE, PAV, and OHE matrices, LD pruning was employed within each vertex after set-specific encoding. All SNPs in a vertex were subjected to LD pruning jointly. No sliding window was applied because most vertices contained a small number of SNPs. Prior to LD pruning, columns in the CORE, PAV, and OHE matrix were filtered by MAF > 0.05. Pruning was performed using the snpgdsLDpruning function of the R package SNPRelate (Zheng et al. 2017) at an LD threshold of 0.6.

LD pruning in the PAV matrix is straightforward and works analogously to LD pruning in a regular SNP matrix of alternative allele counts. LD pruning in the OHE matrix can encounter additional redundancies within markers that came from the same SNP. Particularly, this is the case when either absence or one nucleotide allele is rare. In this case, one of the three markers will not be informative, as it will be (almost) uniform, and the two remaining ones will be (almost) perfect inverses of each other. In these cases, the MAF filter will remove the column for the rare allele. LD pruning will remove one of the two remaining columns, as they are almost perfect inverses of each other, thus being in high LD. What remains is a single column that is almost identical to the original SNP with counts of the rare allele assigned either to absence or the reference allele. Redundancies with neighboring SNPs are eliminated by LD pruning similarly to cases with only biallelic SNPs, the only difference being that estimates of LD are calculated individually for all alleles of a SNP.

For all SNPs that were added to pan-genomic matrices, information of the graph vertex, the respective reference sequence, and, in the case of PAV and OHE, the allele represented by the column was stored with the SNPs. This allows us to distinguish classical SNP markers from PAV and OHE in the final matrices. Matrices were stored in plink2 format with physical positions of SNPs given relative to the reference sequence of the respective vertex.

### Phenotypic data

Phenotypic data were taken from the publicly available 3 k rice genome data (https://snp-seek.irri.org/). The following quantitative traits, which were available for a large number of accessions, were included: culm length (CL), culm number (CN), grain length (GL), grain width (GW), kernel weight (KW), leaf width (LW), leaf length (LL; scored on an ordinal scale from 1 to 5), panicle length (PL), and days to flowering (DF). Phenotypic data for all nine traits were available for a total of 1,603 accessions. For GP, only data from individuals belonging to subpopulations *aus* (*n* = 170), *tropical japonica* (*n* = 233), and *indica* (*n* = 914) were used, while other subpopulations were excluded.

### Model for genomic prediction

For genomic prediction, ridge regression best linear unbiased prediction (rrBLUP) was used. This was one of the first models proposed for genomic selection (Meuwissen et al. 2001). The rrBLUP model can be written as:

$${\varvec{Y}} = 1{\mu} + {\varvec{Zu}} + {\varvec{\varepsilon}}$$

where ***Y*** is an *n*-vector of phenotypes (with n being the number of accessions), 1 is a vector of ones, *µ* is the grand mean, ***Z*** is the n × m genotype matrix (m being the number of selected markers), ***u*** is the vector of marker effects with ***u*** ~ *N*(0, ***I****σ*^*2*^_*u*_), and ***ε*** is the residual error with ***ε*** ~ *N*(0, ***I****σ*^*2*^_*ε*_). The BLUP of the marker effects is given by the ridge regression solution:

$${\varvec{u}} = \left( {{\varvec{Z}}^{\prime } {\varvec{Z}} + {\varvec{I}}\lambda } \right)^{ - 1} {\varvec{Z}}^{\prime } {\varvec{Y}}$$

with *λ* = *σ*^*2*^_*ε*_/*σ*^*2*^_*u*_. Genomic prediction by rrBLUP was implemented using the function mixed.solve() from the R package rrBLUP (Endelman 2011), which estimates *σ*^*2*^_*ε*_ and *σ*^*2*^_*u*_ by REML, solves the mixed model equations, and returns BLUP estimates of marker effects.

For SNPs in BASELINE and PanG-CORE, marker effects represent the additive effect of the alternative allele. For PAV-encoded SNPs, marker effects represent the additive effect of SNP absence. For OHE-encoded SNPs, individual markers represent allele counts for each of the three possible alleles, with effects interpreted as the additive contribution of the respective allele relative to the others.

### Accessing and comparing model accuracies

Model accuracies were assessed by 10 replications of fivefold cross-validation. Genomic predictive ability (GPA) was calculated as the Pearson correlation (*r*) between true and predicted values in the test set over the five folds. The same fold partitions were used for all comparisons. Training was performed separately for the three biggest subpopulations: *aus*, *indica,* and *tropical japonica*. This was done as excessive population structure can bias prediction accuracies, and predictions across subpopulations might not be possible (Werner et al. 2020; Guo et al. 2014). For the *indica*, model prediction accuracies were also assessed within each of the indica subpopulations *indica1A*, *indica1B*, *indica2*, *indica3*, and *admixed indica* by restricting the test set to accessions belonging to the respective subpopulation.

## Results

### Call rates of SNPs in the dispensable genome

A total of 136,974 vertices not present in the Nipponbare (NB) genome were identified across the 15 additional reference sequences (Supplementary Table 1). These non-NB vertices had an average size of 1,588 bp with a total of ~ 2.3 million SNPs previously unidentified by mapping to the Nipponbare (NB) reference sequence (Zhou et al. 2024). This far exceeds requirements for GP. To reduce SNPs to a manageable number while possibly reducing redundancy, we only considered large vertices > 5,000 bp, assuming that SNPs in smaller vertices would more likely be in linkage with neighboring SNPs. The numbers of vertices > 5,000 bp in relation to the overall number of vertices identified in respective reference sequences are given in Supplementary Table 1. The order of reference sequences in the table reflects the order these sequences were used to build the pan-genome in relation to the NB backbone (Zhou et al. 2023). This order does not follow a strict biological reasoning and could be interchanged with any other order without affecting the outcome of SNP selection. Any non-NB vertex is present in at least one of the 15 reference sequences but could be present in all of them (e.g., only absent in NB). For vertices that are present in multiple reference sequences, their assignment to a specific reference merely reflects the order in which reference sequences were compared to NB. That explains why the overall number of vertices (Supplementary Table 1) and SNPs identified within vertices (Table 1) is generally higher for references added earlier. Exceptions exist when a representative of a different subpopulation is added, as for ARC (*aromatic*), NABO *(aus*), and KN (*tropical japonica*) following the more common *indica* accessions.

**Table 1:** Overview of the number of SNPs found within vertices of the pan-genome graph per non-NB reference sequence. For all SNPs, the number of SNPs that do not have a missing value for NB was calculated. Call rate (CR) for SNPs was calculated separately for SNPs that do and do not have a missing value for NB. Subpopulations were assigned according to (Wang et al. 2018)

	Subpopulation of reference	No. of SNPs in vertices > 5,000 bp	No. of SNPs not missing in NB	Mean CR of SNPs missing in NB	Mean CR of SNPs not missing in NB
MH63	*admixed indica*	567,018	442,452	0.52	0.92
ZS97	*indica 1A*	203,761	158,006	0.48	0.9
N22	*aus*	151,180	65,926	0.28	0.78
AZ	*tropical japonica*	73,700	34,172	0.34	0.78
IR64	*indica 1B*	50,611	23,254	0.32	0.78
ARC	*aromatics*	74,021	34,224	0.28	0.73
LM	*indica 2*	56,479	22,494	0.29	0.78
LX	*admixed indica*	34,995	17,084	0.30	0.75
KYG	*indica 3*	29,442	13,690	0.28	0.75
LIMA	*indica 3*	24,340	12,187	0.29	0.77
NABO	*aus*	34,508	16,406	0.27	0.78
PR106	*indica 1B*	11,029	6,625	0.31	0.77
KN	*tropical japonica*	23,899	12,655	0.29	0.77
CM	*subtropical japonica*	13,692	6,075	0.31	0.72
GS	*indica 2*	14,932	7,732	0.25	0.72
Sum/average:		1,363,607	872,982	0.32	0.78

**Fig. 1:**
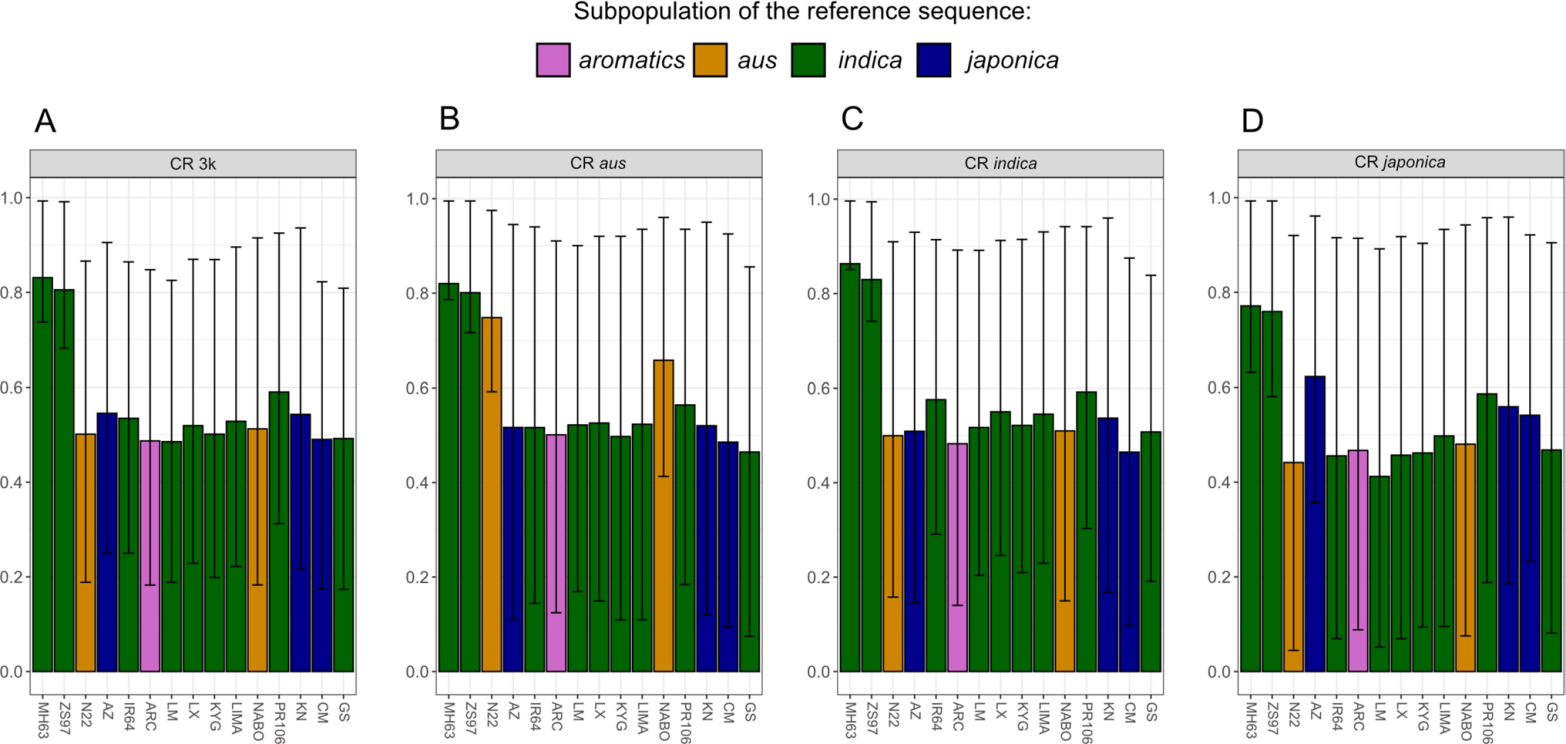
Mean call rates (CR) of SNPs called from non-NB vertices of the pan-genome graph grouped by the reference sequence of the respective vertices. The order of reference sequences (left to right) represents the order in which reference sequences were used to build the pan-genome graph. Colors indicate subpopulation assignment of the reference sequences. **A** CR across all 3 k accessions, **B** CR in *aus* accessions, **C** CR in *indica* accessions, **D** CR in *japonica* accessions. Error bars represent 25th and 75th percentiles

A total of 1,363,607 SNPs were identified within vertices > 5,000 bp (Table 1), and these were queried across the 3 k set of accessions. The average CR in all 3 k accessions was > 0.8 for SNPs in vertices from the first two reference sequences (MH63, ZS97) used to build the graph (Fig. 1A). However, from the 3rd reference sequence onward, CR dropped to < 0.6, with the 25th percentile being < 0.4. Indicating that a large fraction of SNPs newly identified by mapping to novel reference sequences would not have been included in further analysis after applying a strict CR filter. This drop in CR was less extreme in accessions belonging to the subpopulation of the respective reference sequence. For example, SNPs identified first in N22 (*aus*) had an average CR of 0.74 within *aus* accessions but only 0.49 and 0.44 among *indica* and *japonica* accessions, respectively (Fig. 1B–D). Similarly, SNPs from AZ (*tropical japonica*) had an average CR of 0.62 within *japonica* accessions, dropping to 0.50 and 0.51 in *indica* and *aus* accessions, respectively. This pattern was less pronounced for *indica* reference sequences but was still observed for the 25th percentiles, with particularly low CRs being less common in *indica* compared to *aus* and *japonica.*

### Generation of pan-genomic SNP matrices

**Fig. 2:**
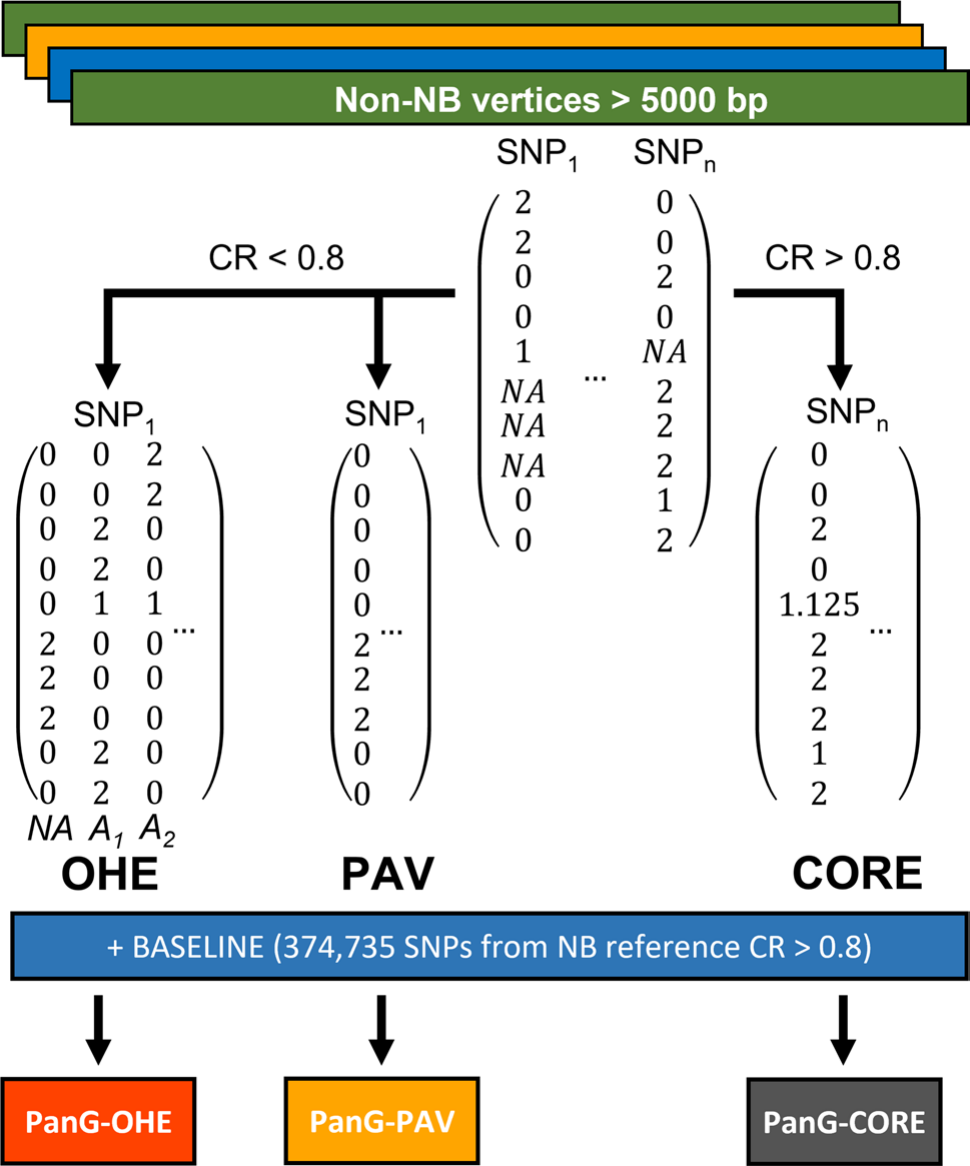
Overview of the process used to generate pan-genomic SNP matrices. Pan-genomic SNP matrices were generated by adding SNPs found in pan-genome graph vertices to the BASELINE SNP matrix. SNPs with call rate (CR) > 0.8, SNPs with CR < 0.8 PAV-encoded, and SNPs with CR < 0.8 OHE were selected for PanG-CORE, PanG-PAV, and PanG-OHE, respectively

Four SNP matrices were created in this study (Fig. 2). The BASELINE SNP matrix was generated by using SNPs identified from mapping short reads to the NB reference and contained 374,735 SNPs after filtering for CR > 0.8 and LD pruning. BASELINE is very similar to the SNP matrices utilized in previous GWAS and GP studies on the 3 k rice genomes project (Rakotondramanana et al. 2022; Yuan et al. 2020; Zhang et al. 2019; Sang He et al. 2022; Tanaka et al. 2021). Other matrices were based on SNPs present in all non-NB vertices selected based on size > 5,000 bp. Of the total 1,363,607 SNPs identified, 708,938 had a CR > 0.8 (Fig. 4) and thus can be considered highly abundant within the rice gene pool despite being identified in non-NB graph vertices. After LD pruning, 46,770 were retained and added to the BASELINE set to create the PanG-CORE SNP set consisting of 421,505 SNPs (Table 2). The third and fourth sets were based on all SNPs with CR < 0.8. SNPs were encoded either by PAV or by OHE. After LD pruning, 166,544 markers were retained for PAV encoding and added to the BASELINE set to create the PanG-PAV set consisting of 541,279 markers. For OHE, every SNP with CR < 0.8 gave rise to three markers representing allele counts of the three possible alleles at the locus. After LD pruning, 288,177 markers were retained. Adding these to BASELINE created the PanG-OHE set consisting of 662,912 markers.

**Table 2:** Overview of the number of markers added to the pan-genomic matrices per non-NB reference sequence in the graph

	Subpopulation of reference	No. of markers added to PanG-CORE (CR > 0.8)	No. of markers added to PanG-PAV (CR < 0.8)	No. of markers added to PanG-OHE (CR < 0.8)
MH63	*admixed indica*	21,957	34,805	71,042
ZS97	*indica 1A*	8,121	14,890	29,588
N22	*aus*	3,709	30,452	48,418
AZ	*tropical japonica*	2,110	13,035	21,772
IR64	*indica 1B*	1,615	8,579	15,087
ARC	*aromatics*	1,917	16,180	25,061
LM	*indica 2*	1,606	11,961	18,965
LX	*admixed indica*	977	6,860	11,059
KYG	*indica 3*	923	5,042	8,402
LIMA	*indica 3*	722	3,874	6,441
NABO	*aus*	1,028	8,056	12,033
PR106	*indica 1B*	353	1,485	2,704
KN	*tropical japonica*	833	5,450	8,265
CM	*subtropical japonica*	451	3,213	4,973
GS	*indica 2*	448	2,662	4,367
Sum:		46,770	166,544	288,177

### Genomic prediction

**Fig. 3:**
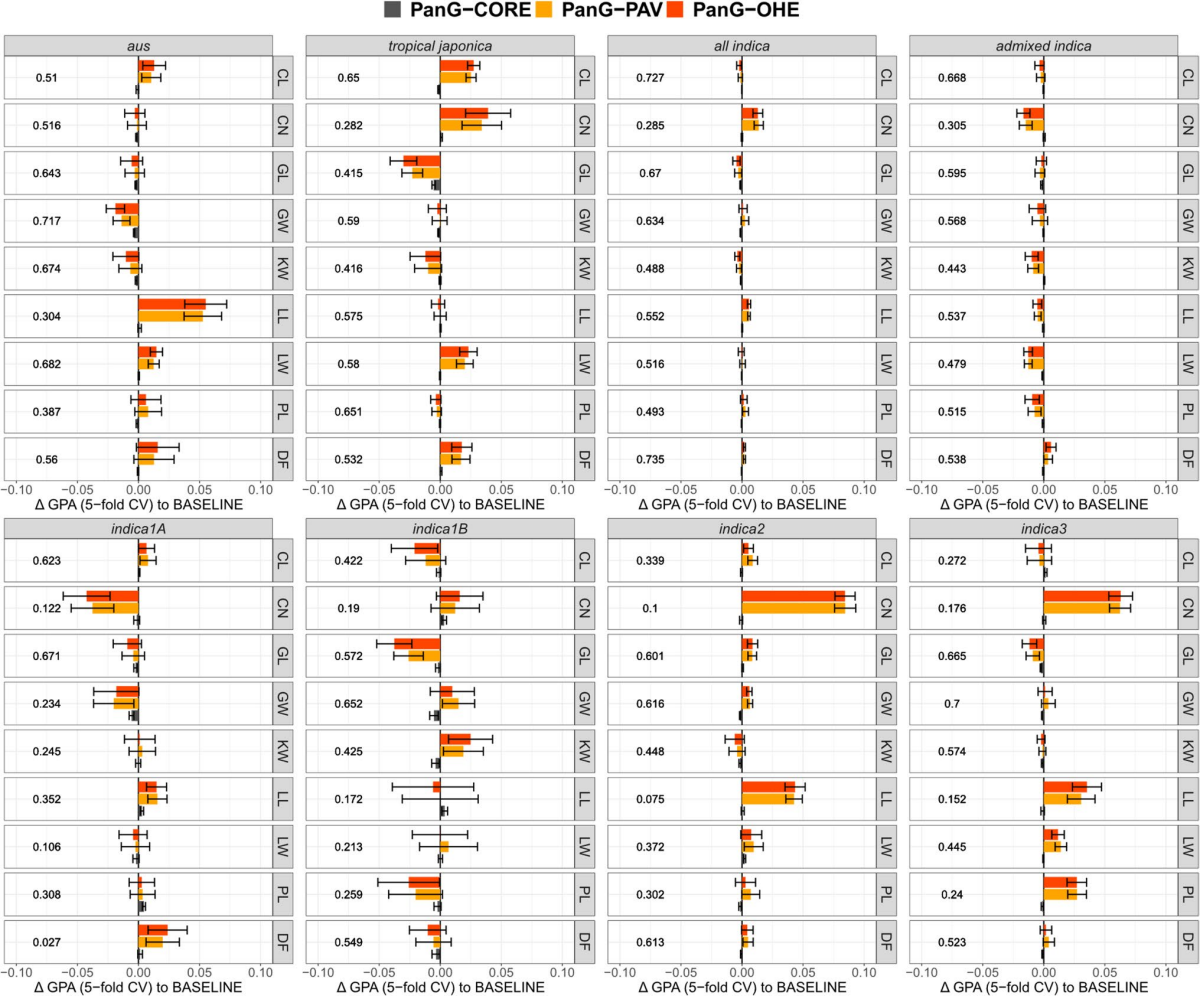
Comparisons of prediction accuracies of genomic prediction (GP) models based on pan-genomic SNP matrices, shown as the change in genomic predictive ability (Δ GPA) relative to the BASELINE model. Bars represent mean ± SD across 10 replications of fivefold cross-validation (CV). Values in the left half of the plot indicate the mean GPA of the BASELINE model. Traits are listed on the y-axis. Colors denote different pan-genomic SNP matrices. Models were trained separately for *aus* (*n* = 170), *tropical japonica* (*n* = 233), and *indica* (*n* = 914) accessions. For indica, accuracies were calculated for all accessions jointly and separately for *indica1A* (*n* = 123), *indica1B* (*n* = 51), *indica2* (*n* = 211), *indica3* (*n* = 243), and *admixed indicas* not assigned to a distinct *indica* subpopulation (*n* = 286)

All four matrices (BASELINE, PanG-CORE, PanG-PAV, and PanG-OHE) were used to train GP models by rrBLUP. Models were trained separately for *aus*, *tropical japonica,* and *indica* accessions to avoid possible bias due to strong population structure effects (Werner et al. 2020; Guo et al. 2014). Since the *indica* subpopulation further stratifies into the minor subpopulations (*indica1A*, *indica1B*, *indica2*, and *indica3),* accuracy estimates for the *indica* models were also calculated for each of these subpopulations, as well as *admixed indicas* separately. *Temperate japonicas* were excluded from our analysis for two reasons. One is their small population size (*n* = 115), and the other is the fact that the phenotypes used were generated during field trials in the Philippines, which is outside the region of adaptation for *temperate japonicas*, making these phenotypes potentially unreliable. The minor subpopulations of *aromatics* and *subtropical japonicas* were also excluded due to insufficient population size to form training sets of reasonable size for GP. Genomic predictive ability (GPA) was estimated by 10 replications of fivefold cross-validation as the Pearson correlation of true and predicted values in test sets. GPA for the BASELINE model varied from as low as 0.027 for DF in *indica2* to as high as 0.74 for DF in *indica* (Fig. 3). Some traits, such as GL, had high GPA (> 0.40) in all subpopulations, but in many instances, GPA for the same trait varied across subpopulations (e.g., LL with GPA = 0.57 in *tropical japonica* and GPA = 0.3 in *aus*; DF with GPA = 0.027 in *indica1A* and GPA > 0.5 in all other subpopulations).

Employing the PanG-CORE SNP matrix in GP generally had negligible effects, with |ΔGPA| never exceeding 0.01 (Fig. 3). Changes in GPA from employing PanG-PAV and PanG-OHE were trait- and subpopulation-specific. Substantial increases in GPA were observed for LL in *aus*; CL, CN, LW, and DF in *tropical japonica*; CN and LL in *indica2*; and CN, LL, and PL in *indica3*. In the majority of cases, changes in GPA were minor. Some substantial decreases in GPA were observed (e.g., GL in *tropical japonica*; GW in *aus*). Overall, accuracy estimates varied substantially in the *indica1A* (*n* = 123) and *indica1B* (*n* = 51) subpopulations, likely due to the limited number of accessions in the test set making these estimates less reliable. The PanG-PAV and PanG-OHE models performed very similarly, with PanG-OHE outperforming PanG-PAV in a few cases (CN and LW in *tropical japonica*; LL in *indica3*), although these differences were small.

The PanG matrices contained a larger number of SNPs compared to BASELINE. To ensure that improvements in GPA were not simply due to the increased number of markers, the number of SNPs in the BASELINE matrix was increased up to 612,540 by employing less strict thresholds during LD pruning. This led to only minor, if any, improvements in prediction accuracy (Supplementary Table 2). Therefore, increases in GPA for PanG-PAV cannot simply be explained by the increased number of model predictors but must be attributed to the alternate selection and encoding strategy.

### Spurious misalignments causing redundancies for high-CR SNPs

**Fig. 4:**
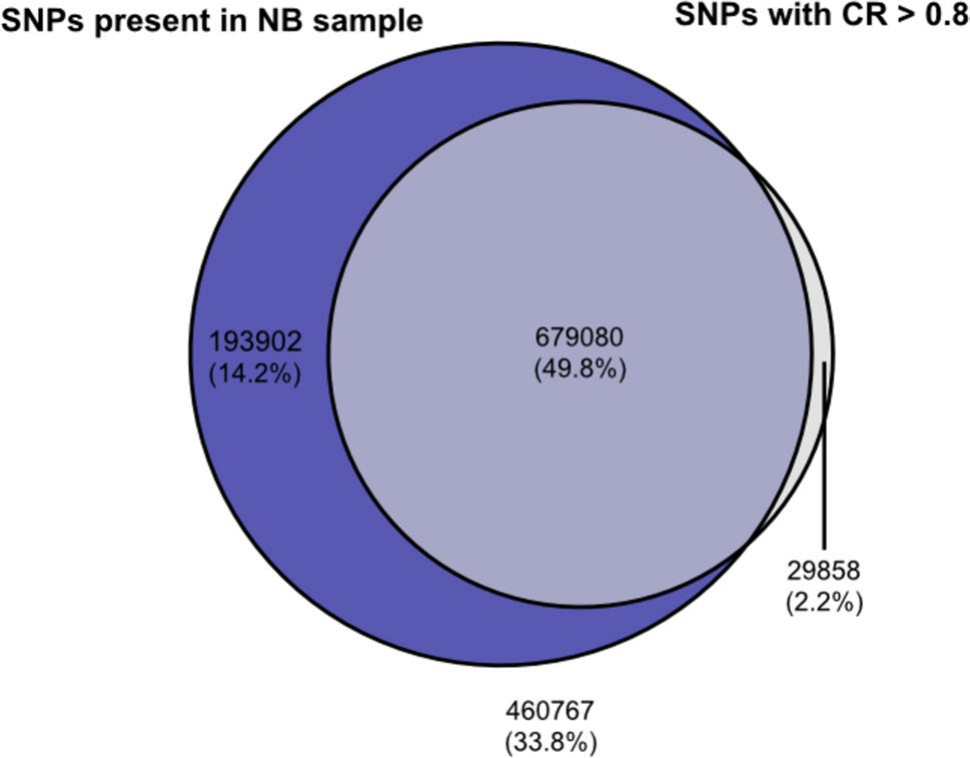
Venn diagram showing the number of pan-genomic SNPs present in the NB sample and SNPs with CR > 0.8

Using pan-genomes for SNP identification is primarily useful for detecting novel SNPs associated with PAV (Hurgobin and Edwards 2017). Misalignments in read mapping are common (Nielsen et al. 2011) and frequently observed in highly repetitive plant genomes (Ribeiro et al. 2015). To investigate why common SNPs of high CR found in the dispensable genome did not improve prediction accuracies, potential erroneous causes for high CR of SNPs were investigated. In the pan-genome graph any non-NB vertex represents a sequence absent from NB. Therefore, no short reads spanning these sequences should be available from the NB sample in the 3 k set, and any of these SNPs should be absent in the NB sample. However, of the 1.36 million SNPs identified in non-NB vertices, 0.87 million were present in the NB sample. Their average CR was 0.78 and thus substantially larger than the average CR of 0.32 for SNPs absent in the NB sample (Table 1). This trend was observed for all SNPs irrespective of the reference sequence they were selected from. A relatively simple explanation for this is that these SNPs resulted from misaligned reads due to high sequence homology. This would be expected for different copies of a sequence affected by copy number variation (CNV). Of all SNPs with CR > 0.8, 95% were detected in the NB sample (Fig. 4), while of all SNPs absent in the NB sample, 94% have CR < 0.8. Given that short reads spanning sequences affected by CNV could unambiguously align to any copy of the respective sequence, one can assume that these high-CR SNPs tend to be redundant with SNPs already contained in the BASELINE matrix.

## Discussion

A single reference sequence does not adequately represent the sequence diversity of a species, and this has spurred efforts to provide additional reference sequences in order to capture the pan-genome for a number of crop species (Zhou et al. 2022; Yan et al. 2023; Li et al. 2023; Jayakodi et al. 2024). Here we utilized the original rice reference sequence of Nipponbare (NB) together with an additional 15 near gap-free reference sequences as our rice pan-genome resource, to which short reads of 3000 gene bank accessions have been mapped as part of the 3 k rice genomes project (Zhou et al. 2020, 2024). In such a resource, it is possible to distinguish a core genome present in all individuals of the gene pool from the dispensable genome present in some but absent in other individuals. The latter is characterized by structural variations (SVs), including CNVs and PAVs. The potential importance of the dispensable genome has been highlighted recently (Zhou et al. 2022; He et al. 2023; Liu et al. 2024; Yan et al. 2023; Yan et al. 2024; Qin et al. 2021). In rice, important QTLs have been discovered in the dispensable genome even before (multiple) reference sequences were available. These include the *Pup1* locus enhancing tolerance to phosphorus deficiency as a result of PAV (Heuer et al. 2009) and the *Sub1* locus, where the presence of a third gene copy conferred submergence tolerance (Xu et al. 2006). The pan-genome resource should improve the power to detect such influential loci within the dispensable genome and to build prediction models that capture their effect. To achieve that goal, it is crucial to optimize the selection of SNPs for GP applications in order to minimize redundancy while maximizing information gain. This has not been done for the rice pan-genome resource. A key objective of our study was therefore to explore different options in producing pan-genome SNP matrices that fit into existing pipelines for GP and capture the novel variation within the dispensable genome.

### SNP selection can improve genomic predictive ability (GPA)

It can be assumed that SNPs from the core genome can be identified by mapping to a linear reference sequence, irrespective of which specific sequence is employed. Therefore, SNPs residing in the core genome will be redundant across all 16 SNP sets employed in this work. SNPs not yet identified by mapping to NB will reside in the dispensable genome and can be captured by focusing on vertices selected from pan-genome graphs. To reduce the SNP set to a manageable size, we only retained vertices > 5,000 bp as a source of SNPs following the rationale that a) variation in smaller stretches of sequence is more likely to be in LD with neighboring SNPs, b) large vertices are more likely within large insertions (relative to NB) and may contain entire genes, therefore capturing causal variation, and c) in a larger sequence containing several SNPs, the absence of all SNPs is more likely to be due to PAV compared to technical error. In fact, a large proportion of SNPs from graph vertices is absent from the majority of samples (Fig. 1). Utilization of SNPs for GP (as well as GWAS) typically relies on selection of SNPs based on high CR and imputation of remaining (rare) missing values. This approach was followed in the assembly of BASELINE and PanG-CORE SNP matrices. BASELINE was limited to SNP draws from the NB SNP set and represents a standard SNP matrix based on the single NB reference, whereas SNPs with CR > 0.8 identified in the dispensable genome were added to BASELINE to form the PanG-CORE matrix. In contrast, the dispensable genome portion added to BASELINE to form PanG-PAV and PanG-OHE matrices was drawn solely from SNPs of CR < 0.8 (Fig. 2).

Comparing these four SNP matrices in GP clearly indicated that putative improvements in GPA over BASELINE were negligible for the PanG-CORE matrix. The only substantial improvements in GPA were observed for the PanG-PAV and PanG-OHE matrices that only included SNPs with a CR < 0.8 (average CR in PanG-PAV = 0.37). To our knowledge, this is the first successful attempt at improving GPA by directly targeting presence–absence variation of SNPs. A previous study explored using failed SNP calls from SNP chip data in canola and maize; however, no improvements compared to only employing regular SNPs were observed (Weber et al. 2023). A possible reason for the failure to increase GPA could be linked to the SNP selection process to establish SNP chips, where candidate SNPs are typically excluded when generating missing calls at high frequency (Ganal et al. 2011; Clarke et al. 2016). This would bias SNP chips toward the core genome. That improvements in our case were only seen for matrices that specifically avoid such a bias may confirm this point. For future SNP chip design for crops with sufficient degrees of PAV, it may be advisable to specifically include a proportion of SNPs with low CR if their presence in the dispensable genome can be confirmed. This was possible in our study through selection of SNPs based on high-quality pan-genome sequences. However, this approach necessitates careful reconsideration of the thresholds applied in CR filtering. It should be noted that, although improvements in GPA from using low-CR SNPs were trait- and subpopulation-specific, these gains result entirely from altered handling of existing data, making them implementable at no additional cost.

### Minimizing errors during SNP selection and imputation

While the approach of handling SNPs by CR filters with the imputation of remaining missing values has been very successful in the past in GWAS (Zhang et al. 2019; Yuan et al. 2020) and GP (Tanaka et al. 2021; Rakotondramanana et al. 2022; Sang He et al. 2022), it assumes that the underlying sequence a SNP came from is present in all individuals and that missing values of that SNP are solely based on errors in SNP calling. This assumption is obviously incorrect for SNPs located in presence–absence variation. Patterns of SNP-missingness were found to be non-random but related to population structure, indicating that absence of SNPs would at least partially retain biologically relevant information **(**Fig. 1**)**, confirming findings by Gabur et al. (2018) and Weber et al. (2023). This highlights the importance of accepting the true absence of SNPs to ensure that SNP sets are representative of the sequence diversity found in the samples. Missing SNP values due to technical errors are indistinguishable from missing values due to PAV (Della Coletta et al. 2021). Strategies to distinguish missing values of biological significance from technical errors have been proposed (Gabur et al. 2018) based on expected allele frequencies in segregating biparental populations and thus cannot be applied to the case at hand. When missing values due to technical errors and biological reasons are indistinguishable, both strategies, either filtering and imputing missing values for SNPs or retaining and encoding missing values as PAV, will produce type I errors, where missing values are falsely imputed or retained. Given the improvements in prediction accuracy observed in this study, we argue that tolerating an unknown rate of type I errors from falsely retaining missing values may be justified in the context of genomic prediction. Future works might try to employ similar principles in GWAS to mine gene banks for beneficial alleles. Potential erroneous missing calls due to technical errors in the genotyping pipeline might cause spurious associations if these are interpreted as true absence of a SNP. Therefore, influential associations between phenotype and absence of SNPs in GWAS may require further independent confirmation. In GP, these issues are less critical because cross-validation already provides independent validations of model performance, which mitigates the impact of individual errors. The main advantage of our approach is therefore to provide more accurate predictions of gene bank accessions, which improves the identification of valuable donors, especially for highly polygenic traits.

### Altered SNP encoding is necessary to integrate SNPs with low CR

As argued above, employing strict CR filters for SNPs found in the dispensable genome removes novel and potentially valuable genotypic information from genotypic matrices. However, as models employed from GP cannot handle missing values, novel methods for encoding SNPs of low CR need to be employed. PAV encoding is a straightforward solution (Weber et al. 2023; Gabur et al. 2018), but it disregards potential nucleotide variation found within dispensable sequences. When accepting absence as a third allele, the variation of a SNP becomes discontinuous. Encoding genotypic states by OHE would result in a four-column matrix including the heterozygous state. However, heterozygosity is negligible in purified rice gene bank accessions (Wang et al. 2018), and heterozygous SNP calls may lead to spurious effect estimates. This was avoided by modified OHE with only three columns per SNP in the matrix, encoding the heterozygous state by allele counts in each of the two nucleotide columns.

One major advantage of this approach is that the resulting matrix is analogous to a regular SNP matrix, allowing for LD pruning using a sliding window and for direct application in existing GP models without further modification. Furthermore, the first marker of the OHE matrix corresponds to the PAV marker, which enables attributing improvements in GP separately to PAV and remaining nucleotide variation. Our results clearly indicated that PAV was the main contributor to improvements in GPA and that further improvements by OHE were minor, if at all detectable.

## Conclusion

In this study, SNPs identified in the dispensable genome by a recently published pan-genomic resource for the 3 k rice genomes project have been used in genomic prediction (GP) of rice gene bank accessions. Improvements to GP accuracies were achieved when adding SNPs of low CR, whereas selection of SNPs from the dispensable genome with CR > 0.8 did not result in any noticeable improvement of GPA. Further comparisons of SNP matrices indicated that encoding as simple PAV was sufficient to capture pan-genome variation and that additional encoding of allelic states within present variants did not further improve GPA. We conclude that the true absence of SNPs due to PAV needs to be considered when handling SNPs identified in the 3 k rice pan-genome resource. Therefore, we advocate that variations of the principles we have utilized in this study could find more widespread employment in GP, both in breeding operations and gene bank phenomics.

## Supplementary Information

Below is the link to the electronic supplementary material.

Supplementary file1 (DOCX 24 KB)

## Data Availability

All data used in this work are publicly available via the 3 k rice genomes project (https://snp-seek.irri.org/), gramene (https://ftp.gramene.org/collaborators/Yong_et_al_variation_dumps/Rice/), and the Rice Population Reference Panel repository (https://yongzhou2019.github.io/Rice-Population-Reference-Panel/). The generated SNP sets, PanG-PAV and PanG-OHE, will be made available upon request in plink2 format with information on the graph vertex and respective reference sequence provided with the SNP names.
