# Supplementary material for: Low-call-rate SNPs and presence–absence variation identified in the rice pan-genome can improve genomic prediction of rice gene bank accessions
Source: Theor Appl Genet. 2025 Nov 7;138(12):295. doi: 10.1007/s00122-025-05080-x (PMC12594682; doi:10.1007/s00122-025-05080-x)
Supplement: Supplementary file 1 — Supplementary file1 (DOCX 24 KB) [file 122_2025_5080_MOESM1_ESM.docx]

**Supplementary Table 1:** Overview of the number of vertices > 5000 bp in the graph (generated by Zhou et al (2023)) per non-NB reference sequence compared to the total number of vertices identified from the respective reference sequence. Order of the sequences (top to bottom) represents the order reference sequences were used to build the graph.

|  | **Subpopulation of reference sequence** | **No. of vertices** | **Total length of vertex sequences [bp]** | **No. of vertices > 5000 bp** |
| --- | --- | --- | --- | --- |
| **MH63** | *admixed indica* | 44,743 | 78,133,762 | 4,175 |
| **ZS97** | *indica 1A* | 14,338 | 25,725,751 | 1,415 |
| **N22** | *aus* | 15,180 | 26,745,825 | 1,584 |
| **AZ** | *tropical japonica* | 7,968 | 13,412,635 | 821 |
| **IR64** | *indica 1B* | 5,575 | 10,023,409 | 653 |
| **ARC** | *aromatics* | 7,500 | 12,308,047 | 751 |
| **LM** | *indica 2* | 8,751 | 11,384,316 | 691 |
| **LX** | *admixed indica* | 6,118 | 7,629,248 | 449 |
| **KYG** | *indica 3* | 3,745 | 5,877,312 | 353 |
| **LIMA** | *indica 3* | 3,151 | 4,564,144 | 264 |
| **NABO** | *aus* | 9,311 | 8,348,824 | 453 |
| **PR106** | *indica 1B* | 1,638 | 1,915,576 | 113 |
| **KN** | *tropical japonica* | 3,727 | 5,011,030 | 316 |
| **CM** | *subtropical japonica* | 3,002 | 3,464,068 | 201 |
| **GS** | *indica 2* | 2,227 | 3,012,310 | 184 |
| **Sum:** | | 136,974 | 217,556,257 | 12,423 |

**Supplementary Table 2:** Genomic predictive ability (GPA) for all traits for the BASELINE models depending on the LD-threshold employed during LD-pruning.

|  | | | **GPA for trait:** | | | | | | | | |
| --- | --- | --- | --- | --- | --- | --- | --- | --- | --- | --- | --- |
| **Subpopulation** | **LD-threshold** | **No. of SNPs** | **Culm length (CL)** | **Culm number (CN)** | **Grain length (GL)** | **Grain width (GW)** | **Kernel weight (KW)** | **Leaf length (LL)** | **Leaf width (LW)** | **Panicle length (PL)** | **Days to Flowering (DF)** |
| *aus* | 0.8 | 374,735 | 0.51 | 0.52 | 0.64 | 0.72 | 0.67 | 0.30 | 0.68 | 0.39 | 0.56 |
| *aus* | 0.9 | 471,388 | 0.51 | 0.52 | 0.64 | 0.72 | 0.68 | 0.31 | 0.68 | 0.39 | 0.56 |
| *aus* | 0.95 | 612,540 | 0.51 | 0.52 | 0.64 | 0.72 | 0.68 | 0.31 | 0.69 | 0.39 | 0.56 |
| *indica1A* | 0.8 | 374,735 | 0.62 | 0.12 | 0.67 | 0.23 | 0.24 | 0.35 | 0.11 | 0.31 | 0.03 |
| *indica1A* | 0.9 | 471,388 | 0.62 | 0.12 | 0.67 | 0.24 | 0.25 | 0.35 | 0.10 | 0.31 | 0.02 |
| *indica1A* | 0.95 | 612,540 | 0.62 | 0.12 | 0.67 | 0.25 | 0.26 | 0.35 | 0.10 | 0.30 | 0.01 |
| *indica1B* | 0.8 | 374,735 | 0.42 | 0.19 | 0.57 | 0.65 | 0.43 | 0.17 | 0.21 | 0.26 | 0.55 |
| *indica1B* | 0.9 | 471,388 | 0.43 | 0.19 | 0.57 | 0.65 | 0.42 | 0.18 | 0.21 | 0.25 | 0.54 |
| *indica1B* | 0.95 | 612,540 | 0.43 | 0.19 | 0.57 | 0.66 | 0.41 | 0.18 | 0.21 | 0.24 | 0.54 |
| *indica2* | 0.8 | 374,735 | 0.34 | 0.10 | 0.60 | 0.62 | 0.45 | 0.08 | 0.37 | 0.30 | 0.61 |
| *indica2* | 0.9 | 471,388 | 0.34 | 0.10 | 0.60 | 0.62 | 0.45 | 0.08 | 0.38 | 0.31 | 0.61 |
| *indica2* | 0.95 | 612,540 | 0.35 | 0.09 | 0.59 | 0.62 | 0.45 | 0.09 | 0.38 | 0.31 | 0.62 |
| *indica3* | 0.8 | 374,735 | 0.27 | 0.18 | 0.67 | 0.70 | 0.57 | 0.15 | 0.44 | 0.24 | 0.52 |
| *indica3* | 0.9 | 471,388 | 0.28 | 0.18 | 0.67 | 0.70 | 0.57 | 0.16 | 0.45 | 0.24 | 0.52 |
| *indica3* | 0.95 | 612,540 | 0.27 | 0.18 | 0.67 | 0.70 | 0.58 | 0.16 | 0.45 | 0.24 | 0.52 |
| *all indica* | 0.8 | 374,735 | 0.73 | 0.29 | 0.67 | 0.63 | 0.49 | 0.55 | 0.52 | 0.49 | 0.74 |
| *all indica* | 0.9 | 471,388 | 0.73 | 0.28 | 0.67 | 0.64 | 0.49 | 0.55 | 0.52 | 0.49 | 0.73 |
| *all indica* | 0.95 | 612,540 | 0.73 | 0.28 | 0.67 | 0.64 | 0.49 | 0.55 | 0.52 | 0.49 | 0.73 |
| *admixed indica* | 0.8 | 374,735 | 0.67 | 0.30 | 0.59 | 0.57 | 0.44 | 0.54 | 0.48 | 0.52 | 0.54 |
| *admixed indica* | 0.9 | 471,388 | 0.67 | 0.30 | 0.59 | 0.57 | 0.44 | 0.54 | 0.48 | 0.52 | 0.54 |
| *admixed indica* | 0.95 | 612,540 | 0.67 | 0.30 | 0.59 | 0.57 | 0.44 | 0.54 | 0.48 | 0.52 | 0.53 |
| *tropical japonica* | 0.8 | 374,735 | 0.65 | 0.28 | 0.42 | 0.59 | 0.42 | 0.58 | 0.58 | 0.65 | 0.53 |
| *tropical japonica* | 0.9 | 471,388 | 0.65 | 0.29 | 0.42 | 0.59 | 0.42 | 0.57 | 0.58 | 0.65 | 0.53 |
| *tropical japonica* | 0.95 | 612,540 | 0.65 | 0.29 | 0.43 | 0.60 | 0.42 | 0.57 | 0.58 | 0.65 | 0.53 |
